# Supplementary material for: Facile Fabrication of TiO2 Quantum Dots-Anchored g-C3N4 Nanosheets as 0D/2D Heterojunction Nanocomposite for Accelerating Solar-Driven Photocatalysis
Source: Nanomaterials (Basel). 2023 May 6;13(9):1565. doi: 10.3390/nano13091565 (PMC10180858; doi:10.3390/nano13091565)
Supplement: Supplementary file 1 [file nanomaterials-13-01565-s001.zip › nanomaterials-2341073-supplementary.pdf]

Supporting Information

for

Facile Fabrication of TiO_2 Quantum Dots-Anchored g-C_3N_4 Nanosheets as 0D/2D Heterojunction Nanocomposite for Accelerating Solar-Driven Photocatalysis

Jin-Hyoek Lee [1,†], Sang-Yun Jeong [1,†], Young-Don Son [2] and Sang-Wha Lee [1,*]

[1] Chemical and Biological Engineering Department, Gachon University, , Seongnam-si 13120, Republic of Korea; 71in.cmsal@gmail.com (J.-H.L.); wjdtkddbs762@naver.com (S.-Y.J.)
[2] Department of Biomedical Engineering, College of IT Convergence, Gachon University, Seongnam-si 13120, Republic of Korea; ydson@gachon.ac.kr
* Correspondence: lswha@gachon.ac.kr; Tel.: +82-10-2635-5360
† These authors contributed equally to this work.

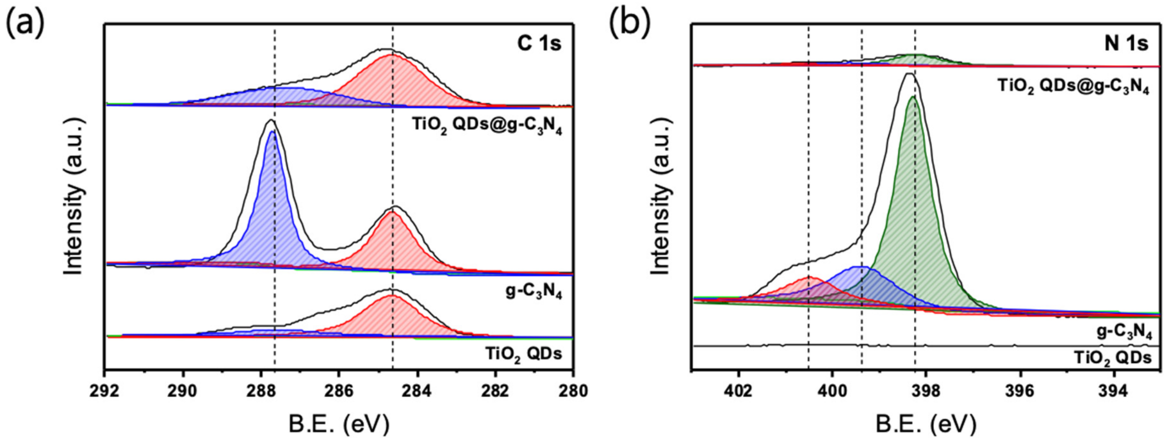

Figure S1. Core-level spectra of TiO_2 QDs, g-C_3N_4 and TiO_2 QDs@g-C_3N_4 by XPS (a) C 1s, (b) N 1s.

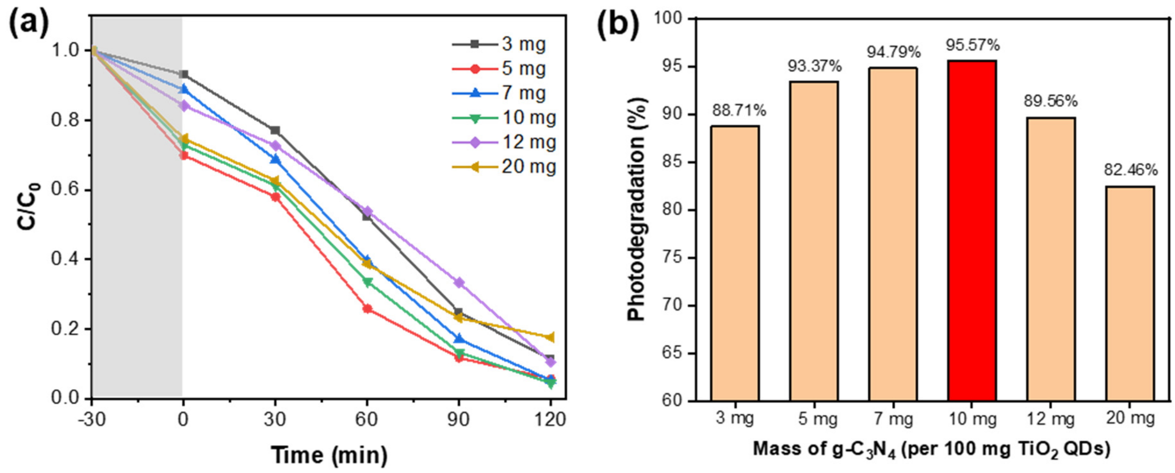

Figure S2. Photodegradation rate of MO dye over the TiO_2 QDs@g-C_3N_4 with different g-C_3N_4 contents under simulated solar light.

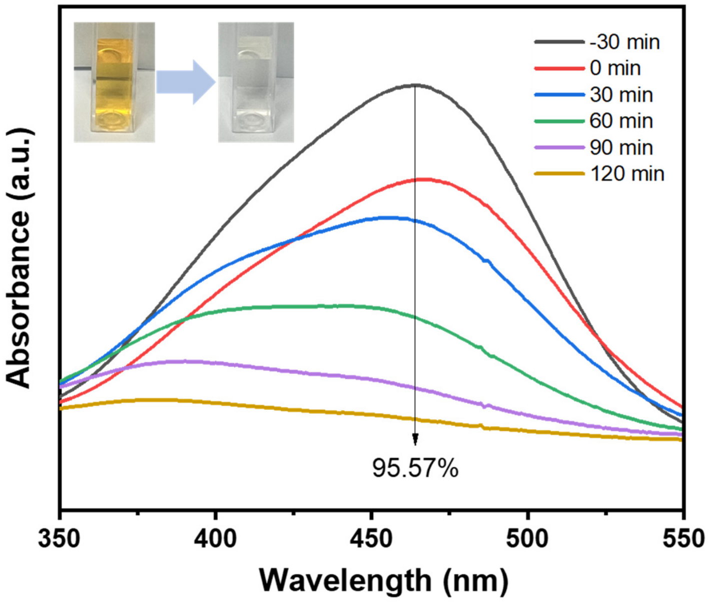

Figure S3. UV-absorbance of MO dye over TiO_2 QDs@g-C_3N_4 under simulated solar light.

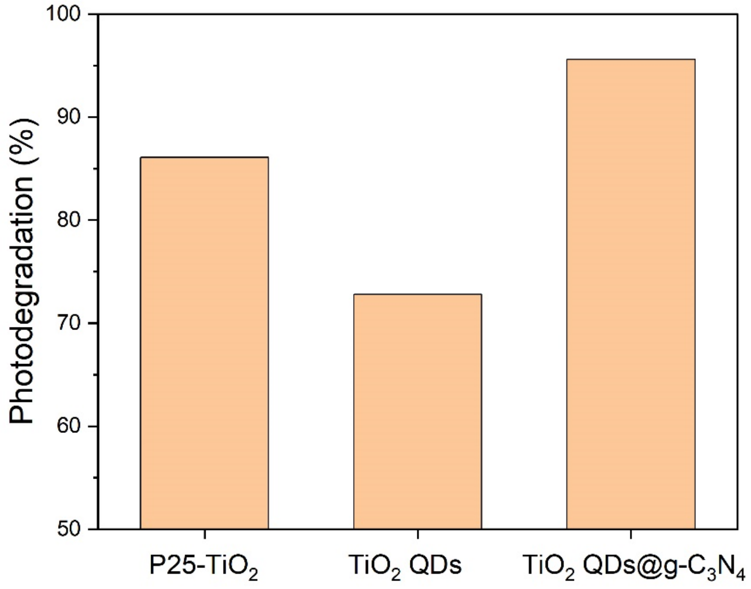

Figure S4. The comparison of photodegradation over as-prepared samples after 120 min irradiation.

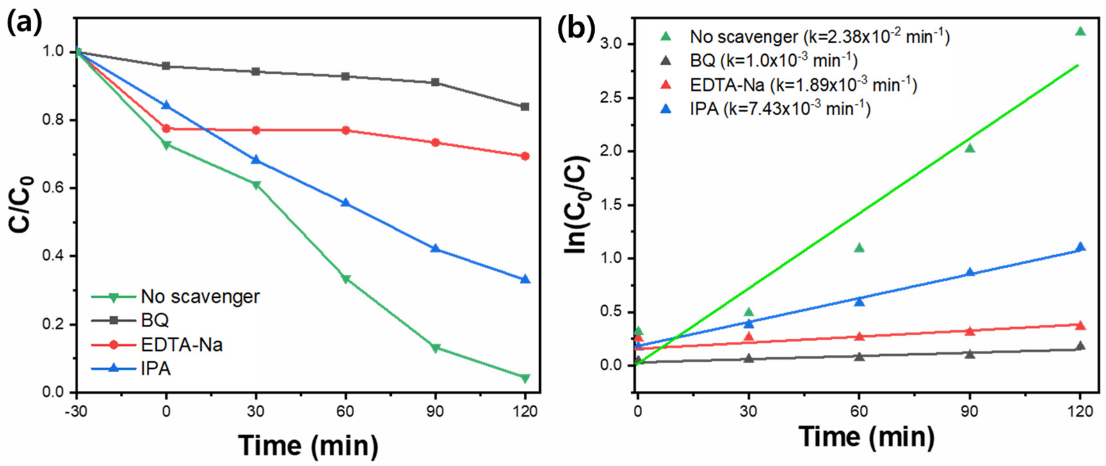

Figure S5. (a) Photodegradation rate of MO dye over the TiO_2 QDs@g-C_3N_4 in the presence of scavengers, (b) Kinetic plot for the photodegradation of MO in (a).

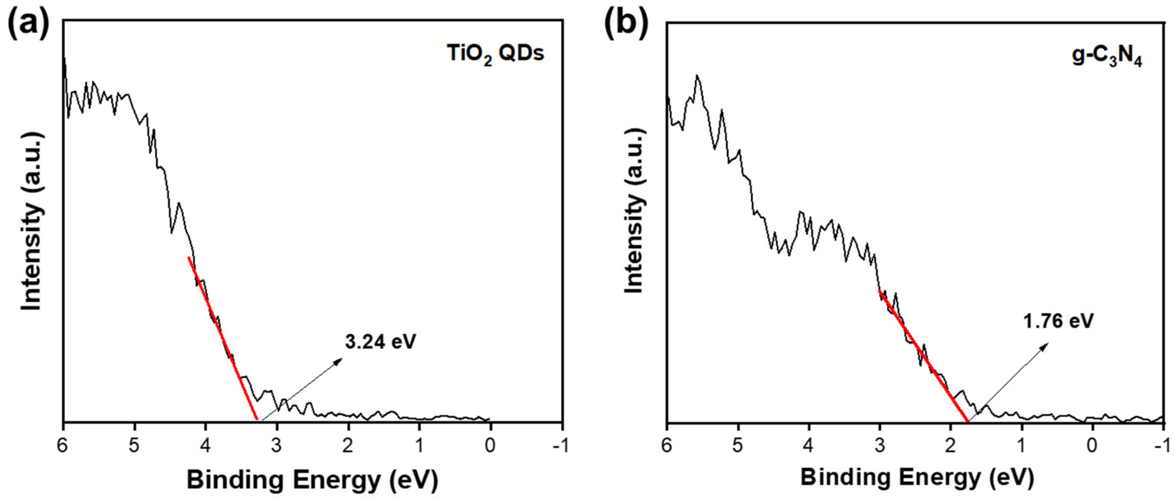

Figure S6. Low-energy XPS VB spectra of (a) TiO_2 QDs, (b) g-C_3N_4 samples.

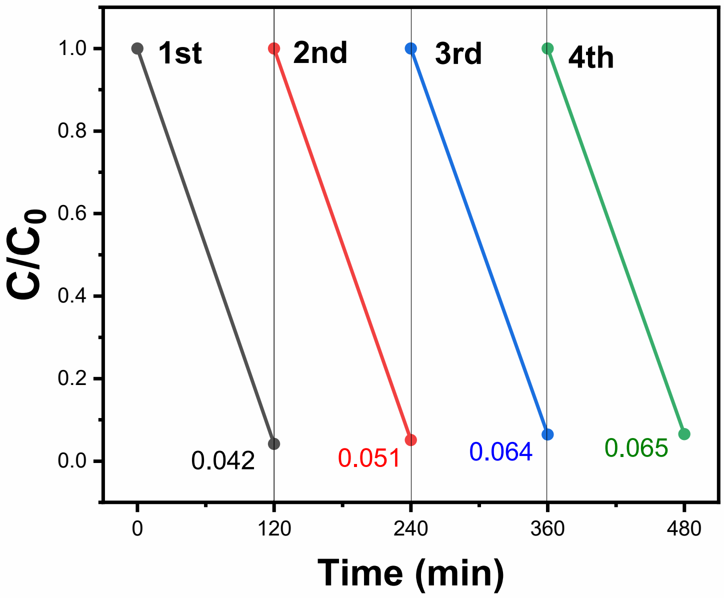

Figure S7. The degradation rate of MO dye after 120 min irradiation using the TiO_2 QDs@g-C_3N_4 photocatalyst that was recycled four times.

Table S1. Elemental composition of TiO_2 QDs@g-C_3N_4 by XPS analysis

Elemental	TiO_2 QDs@g-C_3N_4	
	at. %	wt. %
C	41.55	26.39
N	4.40	3.26
Ti	14.97	37.25
O	39.08	33.10

Table S2. Elemental composition of g-C_3N_4 by XPS analysis

Elemental	g-C_3N_4
	at %
C	51.39
N	42.52
O	6.08
